# Supplementary material for: Autoprobiotics in the Treatment of Patients with Colorectal Cancer in the Early Postoperative Period
Source: Microorganisms. 2024 May 13;12(5):980. doi: 10.3390/microorganisms12050980 (PMC11124500; doi:10.3390/microorganisms12050980)
Supplement: Supplementary file 1 [file microorganisms-12-00980-s001.zip › microorganisms-2908179-supplementary.pdf]

Supplementary Materials: Figure S1

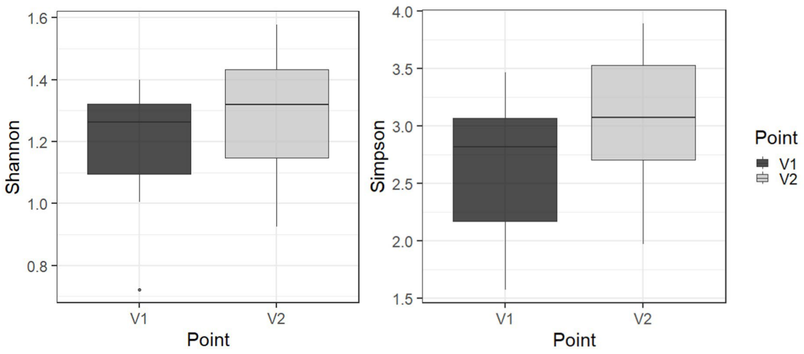

A

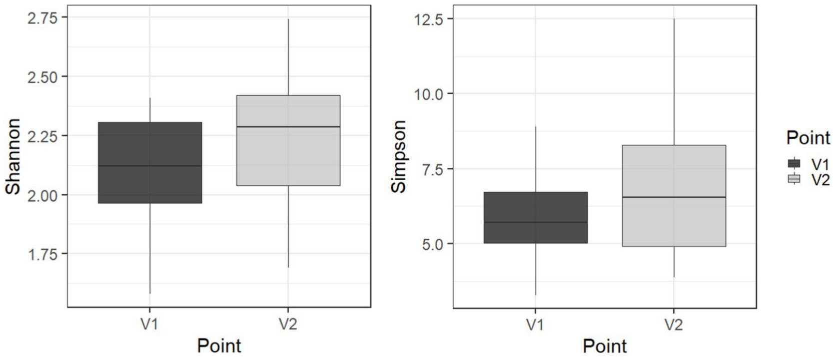

B

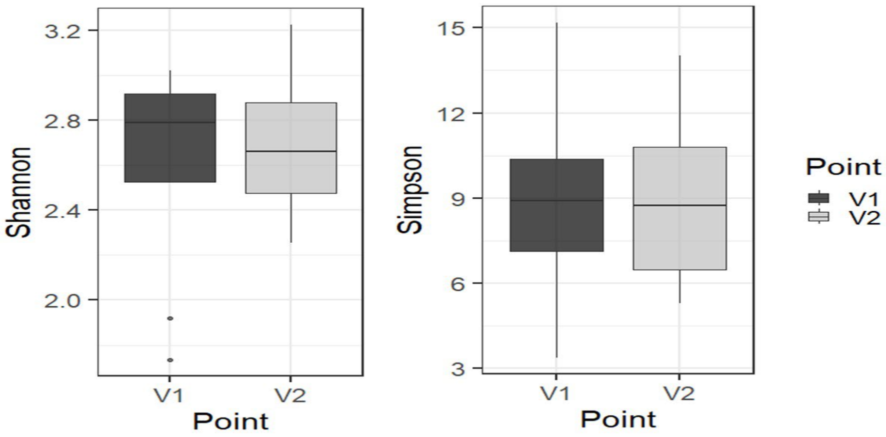

Figure S1. Alpha-biodiversity before and after therapy in the levels of phylum (A), family (B) and genus (C).
